# Who are you, subdistal appendages of centriole?

**DOI:** 10.1098/rsob.180062

**Published:** 2018-07-25

**Authors:** Rustem Uzbekov, Irina Alieva

**Affiliations:** 1 Faculté de Médecine, Université de Tours, 10 Boulevard Tonnellé, 37032 Tours, France; 2 Faculty of Bioengineering and Bioinformatics, Moscow State University, Leninskye gory 73, 119234 Moscow, Russia; 3 Belozersky Institute of Physico-Chemical Biology, Moscow State University, Leninskye gory 1-40, 119992 Moscow, Russia

**Keywords:** centriole, distal appendages, subdistal appendages

## Abstract

This review summarizes data that assign morphological, biochemical and functional characteristics of two types of structures that are associated with centrioles: distal appendages and subdistal appendages. The description of centriole subdistal appendages is often a matter of confusion, both due to the numerous names used to describe these structures and because of their variability among species and cell types. Thus, we have summarized our current knowledge in this review. We conclude that distal appendages and subdistal appendages are fundamentally different in composition and function in the cell. While in centrioles there are always nine distal appendages, the number of subdistal appendages can vary depending on the type of cells and their functional state.

## 1. Introduction

The fact that the centrosome is a complex organelle consisting of many components is today generally accepted [1–4]. The ultrastructural features of these components were described in detail in the middle of the last century. At the beginning of the cell cycle, the centrosome contains two centrioles surrounded by a pericentriolar material, the protein composition of which is now intensively studied [5]. The two centrioles in the centrosome differ in age, functional activity in the cell and the presence of additional structures associated with their surface. The older centriole, which is called the mother, arose at least one cell cycle earlier than the second, which is called the daughter centriole. Centrioles are cylindrical polar structures built of microtubules (MT). At the proximal end of the centriolar cylinder is the minus and at the distal end is the MT plus-end. The distal end of the mother centriole can also grow a primary cilium. In the process of centriole duplication, which begins in the G_1_-phase of the cell cycle [6,7], the formation of new young centrioles—procentrioles occurs on both pre-existing centrioles near their proximal ends. Further, the distal surfaces of mother centrioles are associated with two types of outgrowths, the distal and subdistal appendages. This review focuses on subdistal appendages.

In the past, many cellular components have been simultaneously described by several researchers and given, respectively, different names. In addition, the same component sometimes has intrinsic variations in different cells, which may also lead to terminological confusion. Over time, most of the terms were unambiguously defined. But even now, at the beginning of the twenty-first century, there are terminological discrepancies that complicate the interpretation of the results obtained by modern imaging methods.

It is generally believed that the morphological components of the centrosome are well described and the discrepancies in their definition have long since been eliminated. However, it turns out that in fact it is not so. Different researchers dealing with biology of the centrosome have different ideas about what a subdistal appendage is. Numerous discrepancies found in modern literature motivated us to write this review, the purpose of which is to clarify numerous terms proposed in the literature to describe two types of outgrowths associated centriole cylinders: distal and subdistal appendages.

## 2. ‘Massules’, ‘subdistal appendage’, ‘distal appendage’, ‘pericentriolar satellite’—who is who on the centriole wall?

To give a definitive answer to the question what a subdistal appendage eventually is, it is necessary to make a short digression into the history of the centriole studies, and to define the terms such as ‘appendages’, ‘distal appendages’, ‘subdistal appendages’, ‘satellites’ and ‘pericentriolar satellites’.

**Figure 1.**
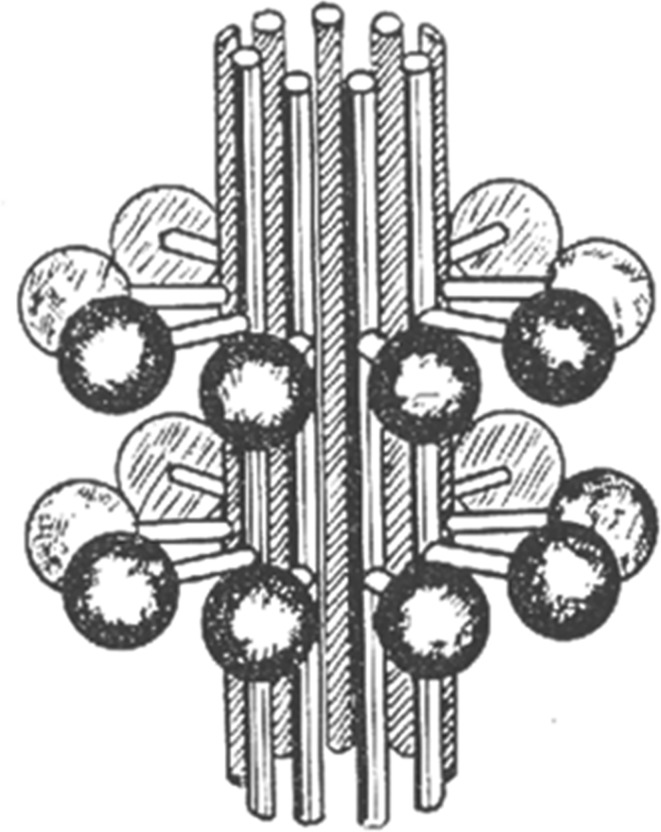
Bessis & Breton-Gorius [8] discovered that the surface of leucocyte centrioles was often covered with growths, consisting of a stem with a round cap, which these authors called ‘massules’.

The first volumetric model of the centriole, on which the outgrowth appeared on the surface of a centriolar cylinder, was published by Bessis & Breton-Gorius in 1958 [8] (figure 1). This was not a 3D reconstruction, but a drawing made by the authors on the basis of their own observations. The authors depicted a centriole which it contained two groups of nine appendages connected with centriole surface, which the authors called ‘massules’.

**Figure 2.**
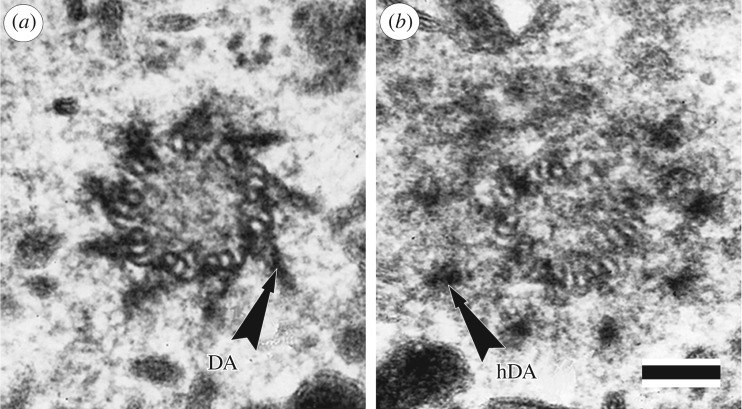
Two consecutive ultrathin sections through the distal part of mother centriole in the cell of porcine kidney embryo cell line. (*a*) Distal appendages (DA) are always present in an amount of 9—one for each triplet (doublet) of MT of the mother centriole. (*b*) Heads of the distal appendages (hDA) are located more distally in comparison with points of attachment of the distal appendages to the surface of the centriolar cylinder and therefore are detected on the next ultrathin section. In the view from the proximal end of the centriole, the MT triplets are twisted counterclockwise, distal appendages are twisted in the opposite direction (clockwise). Scale bar, 100 nm. From [9].

In this figure, both groups of appendages are equidistant from the ends of the centriolar cylinder (it was not yet known that centrioles have proximal-distal polarity). Later, one of these groups, which is located at the distal end of the centriole, was renamed *distal appendages*, or *appendages* (figure 2). There are also other terms in literature that were used to refer to similar structures on basal bodies. For example, the distal appendages were called *satellites* by Szöllösi [10], *alar sheets* by Anderson [11] and *transitional fibres* by O'Hara [12]. However, in this paper we will mainly talk about the structures associated with centrioles of the centrosome, but not basal bodies.

The second group of outgrowths on the surface of the centriole, which are located closer to the central part of the centriolar cylinder, were originally named *pericentriolar satellites* [13], *satellites* [14], *radial arms* [15] and, finally, *subdistal appendages* [16]. The term subdistal appendages [16,17] seems to us less successful, since by the time of its appearance it was already known that subdistal appendages can be located along the entire length of the centriolar cylinders [18]. However, since at present this term is the most common, we will use it in our review.

Although only a hypothetical scheme, the ideas proposed by Bessis and Breton-Gorius in 1958 [8] (figure 1) proved to be tenacious and are regularly reproduced in the diagrams given in modern articles and reports at scientific conferences and unfortunately even in textbooks (for example, Alberts *et al*.'s figs 16–48 [19]). According to these schemes, the centriole is depicted with two rows of outgrowths (nine outgrowths in each); while in each row both subdistal appendages and distal appendages are located on the same level. Sometimes even specialists draw diagrams, where these structures look almost identical [20].

**Figure 3.**
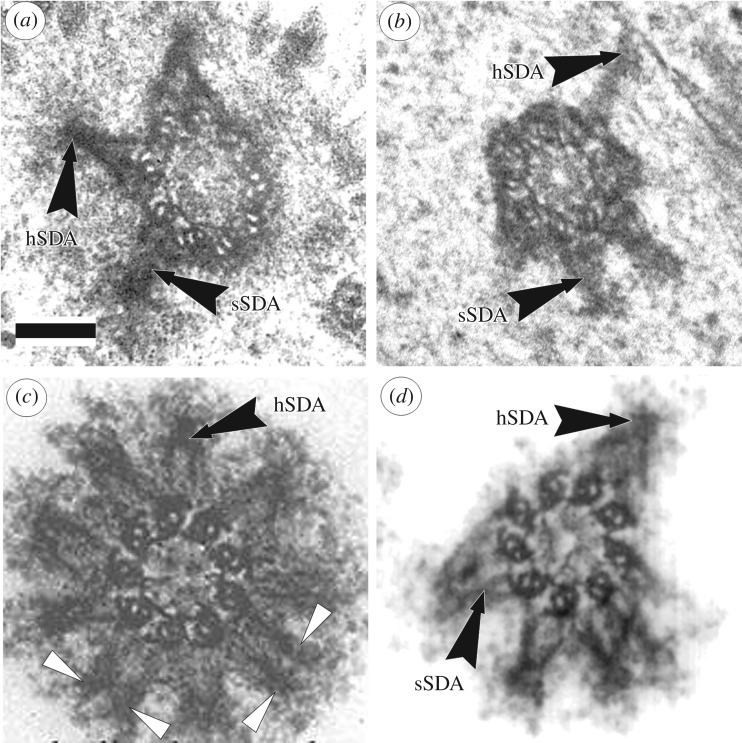
Ultrathin sections through the subdistal part of mother centriole in different types of cells. (*a*) Cell with three subdistal appendages from epithelial pig kidney (PK) embryo cell [9], conical form of subdistal appendages stem (sSDA) with spherical heads (hSDA) are visible. Each subdistal appendage based on 2 or 3 centriolar MT triplets. (*b*) Cell with four subdistal appendages from pig oviduct cell [4]; (*c*,*d*) cells with nine and six subdistal appendages from KE-37 human cell line [16]. Arrowheads show cylindrical subdistal appendages without visible heads and based on 1 MT triplet of centriolar cylinder. In (*a*,*c*,*d*) the triplets are twisted clockwise, hence the view from the distal of the centriole, for photo ‘*b*’ the triplets are twisted counterclockwise, hence the view from the proximal end of the centriole. Scale bar, 100 nm.

One particular paper played a decisive role in spreading this perception. In 1992, *Journal of Structural Biology* published the work with stunningly beautiful photos of centrioles [16], several images of which the publishers put on the cover. In one of the photos, the number of subdistal appendages was 9, although even in this photo it can be seen that part of the subdistal appendages have their base on two triplets of the centriole (figure 3*c*). Later this photo was included in several centrosome reviews [3,17,21–23].

In their article, the authors explicitly state: ‘The number, thickness, and distribution of the subdistal appendages can vary significantly from one centrosome to the other in the same preparation’ [16]. Indeed, we can see photographs of centrioles with 4, 6, 8, 9 subdistal appendages (for example figure 3*c*,*d*) in this article. One should also take into account the fact that isolated centrioles were studied in the work, whose morphology changes under the influence of experimental procedures used to extract centrosomes from cells—use of inhibitors of cytoskeleton components (Nocodazole, Cytochalasin), low temperatures and ultracentrifugation. In particular, in a number of cases, the heads of subdistal appendages are lost, which leads to the loss of their conical shape due to the divergence of parts of the stem associated with each of the triplets (figure 3*c*).

In conclusion, the confusion with the names of the initially identified centriole structures, characteristic for the initial period of electron microscope research, was gradually eliminated and common terms were agreed on. However, from all the terminological diversity in modern literature there is one controversial term—subdistal appendages (pericentriolar satellites)—which is still treated differently by different researchers.

The most common misconception in the field is that each of the subdistal appendages is associated with one triplet of a centriolar cylinder and that; hence the number of subdistal appendages is always nine. Such an opinion is reflected in numerous publications on which, like Bessis & Breton-Gorius [8], the authors depict or describe nine subdistal appendages [16,19,21,23–29]. However, for the sake of justice, it should be noted that in Paintrand *et al*. [16], along with the assertion that subdistal appendages in KE37 cells are always exactly nine, there is a reservation that the number of these structures may be different in other types of cells. The earlier work of the same laboratory [15] demonstrated that the number of subdistal appendages (radial arms in this paper) never exceeded six and they were not located symmetrically upon the centriole.

We fully subscribe to this statement and in this review we present data that distal appendages and subdistal appendages (they are also called ‘pericentriolar satellites’ in other publications) are morphologically different structures having different protein composition and functional purpose.

## 3. Morphological differences between distal appendages and subdistal appendages

The centriole structure proposed by Bessis & Breton-Gorius [8] (figure 1) assumes that both groups of structures connected to the centriole have identical morphology—they have a thin stem and a large round head. However, at that time the methods of sample preparation for electron microscopic analysis were imperfect. The improvement in both specimen fixation and staining methods for electron microscopy [30–32] as well as the more recent development of cryo-electron microscopy [33] have made it possible to refine the description of the ultrastructure of centrioles and basal bodies, including distal and subdistal appendages (figures 2 and 3).

Distal appendages (figure 2) in electron microscopic images look like ‘blades of a turbine’. They are always located centrally symmetrically at the distal end of the cylinder of the mother centriole, their number is constant—they are always nine per centriole. Each distal appendage is associated with only one of the centriole triplets and departs from the centriole surface at an angle of about 50 degrees. Distal appendages are twisted in the opposite direction to the twist of the centriole triplets, that is, counterclockwise, when viewed from the distal end of the centriole. Distal appendages are always present on centrioles in a more or less pronounced form, they are found both on interphase centrioles and on mitotic ones [34]. Distal appendages appear on the centriole during second cell cycle after its ‘birth’, when this centriole becomes the mother and acquires the ability to form the primary cilium.

Subdistal appendages with their base are associated with 2–3 triplets of centrioles and move away from its surface at a right angle (figure 3). Subdistal appendages in the classical form represent a two-component formation, consisting of a rounded head and a conical, sometimes striated stem (figure 3). Figures 2*a* and 3*a* show distal appendages and subdistal appendages in cells of the same PK cell line. Their morphology excludes the possibility of confusing them for the same structure, and further study of their behaviour in the cell only confirms this fact.

Unlike the distal appendages, which are constant in number and structure, subdistal appendages can change morphologically after some influences we will mention below, and are distinguished by variability of form in specialized cells of some organisms. For example, the stem striation can practically disappear, and the stem itself can change shape in the axolotl blood cells [18]. The study of endotheliocytes in intact zones, as well as in fibrous and atheromatous areas of the artery obtained from autopsy material of adults, showed that the form of subdistal appendages was different: in four of the five types of cells identified by the authors, subdistal appendages had a conical shape, and to their heads MT fit, and in cells of one group of the body, subdistal appendages were a complex of fibrils of constant thickness [35,36].

**Figure 4.**
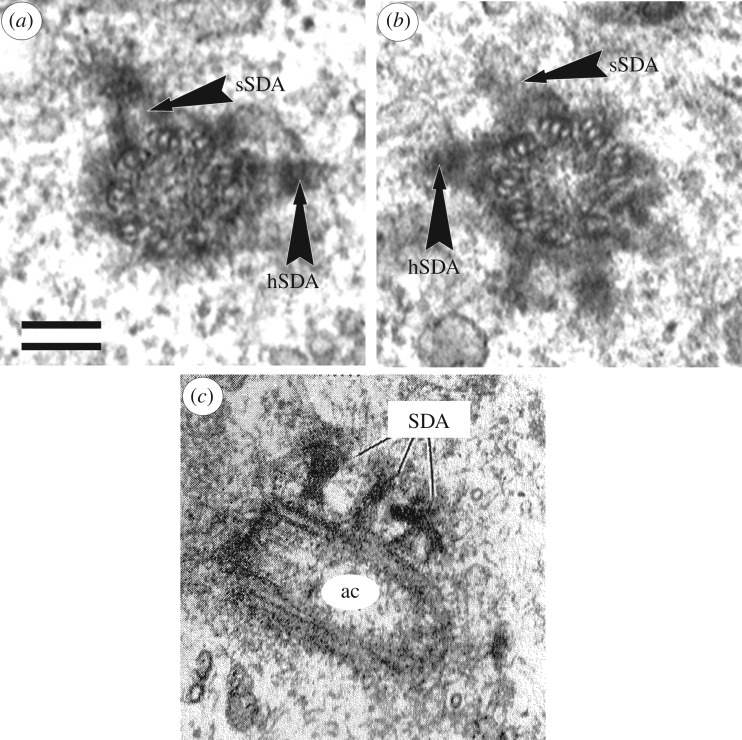
Subdistal appendages can be on the different distance from distal end of centriole. (*a*,*b*) Two consecutives serial cross sections of distal part of mother centriole in the cell of BHK-21 (baby hamster kidney cell line), five subdistal appendages with well visible stem (sSDA) and head (hSDA) parts placed in ‘two flours’ (from [4]). (*c*) The view from the proximal end of the centriole (the MT triplets are twisted counterclockwise); near longitudinal section of active (mother) centriole (ac) from axolotl cell, subdistal appendages (SDA) placed in ‘three flours’ (from [18]). Scale bar, 100 nm.

The location of the subdistal appendages on the centriole wall is not tied to its distal end and can be very different—several subdistal appendages may be located on the same level (figure 3) or can be spaced along the length of the centriolar cylinder (figure 4).

De Harven & Dustin in 1960 [37] first noted that subdistal appendages are detected only on interphase centrioles and never on mitotic ones. A detailed ultrastructural study showed that subdistal appendages (in the author's terminology—pericentriolar satellites) disappear during the pre-mitotic G2 phase of the cell cycle and reappear on centrosomes at the beginning of the post-mitotic G1 phase of the cell cycle [34].

## 4. How many distal appendages and subdistal appendages can be located on one centriole?

Unlike distal appendages, the number of subdistal appendages is variable; it depends on a number of different factors—the type of cell, its functional state, external influences (including hormonal ones) and even the age of the cell. Subdistal appendages may be completely absent in some types of cells [38–43] or there may be even more than nine per centriole [18,35,36].

A change in the functional activity of the centrosome (for example, activation of the centrosome's MT-nucleating capacity by certain metabolic inhibitors) leads simultaneously to an increase in the average number of subdistal appendages [44,45]. In endothelial cells, the number of subdistal appendages on the mother centrioles of human artery cells is very rapidly increased after thrombin treatment [46]. If normal to the centriole of endotheliocytes is present from one to four subdistal appendages (three subdistal appendages for 60% cells), then after 5 min of exposure to thrombin, the number of subdistal appendages grows to 3–7 (four and more subdistal appendages for 70% cells), with no centrioles having less than three subdistal appendages. However, when thrombin was exposed to human vein endotheliocytes, this effect was not observed and the number of subdistal appendages remained the same. In both untreated and treated with thrombin cells, the number of subdistal appendages varied from 3 to 6 (in majority of cells 4 or 5) [46].

In human endotheliocytes, the mother centriole is able to carry an extremely variable number of subdistal appendages from 2 to even 12 per centriole [35,36].

In most cell types, subdistal appendages are only found on a mature mother centriole [47]. However, the presence of these structures on both centrioles is described in embryonic fibroblasts [9] and neutrophils [48], but even in these cases, the number of subdistal appendages on the mother centrioles is always greater than on the daughter centrioles [48].

No one now doubts the correctness of the hypothesis of Henneguy [49] and Lenhossék [50] about the homology of the cilia and flagella basal bodies and centrioles. Subdistal appendages of centrioles are, likewise, homologous structures of the basal feet found on basal bodies. There is one or two basal feet per each basal body [51] and they determine the direction of the ciliary beating [52]. In many cell cultures, the number of subdistal appendages is also 1 or 2. In the case of distal appendages, they are always exactly 9, and they are called the same called the same for both the centriole and the basal body [12]. However, for some objects, the distal appendages all still have their own names, for example, in the basal bodies of *Chlamydomonas* they were called [53] and continue to be called transitional fibres [54].

## 5. Functional and immunochemical differences subdistal appendages and distal appendages

Distal and subdistal appendages are not only morphologically different, but also functionally diverse. The first attaches the centriole to the cell membrane during the formation of the cilium, while the latter represent miniature centres of microtubule nucleation—MT are actively nucleated from their heads (and they are attached for a while) [34,45].

Proteins of the distal and subdistal appendages have been actively studied since the beginning of this century, immunochemical studies have allowed us to compile a unique map of protein localization in various centrosomal components, including subdistal appendages [4,55]. γ-tubulin—the protein that directly participates in the nucleation of MT on centrosome—is present in the heads of subdistal appendages [56]. Proteins involved in the process of stabilization and anchorage of MT into subdistal appendages and in the regulation of the maturation of centrioles were found in subdistal appendages too [57,58]. Later, several other proteins were also classified as subdistal appendages proteins: ε-tubulin [59], CEP110 = centriolin [60,61], CEP170 [62], CC2D2A [63]. Their interactions were described and the sequence of their appearance on the centriole was established. It turned out that ninein binds to the centriole at its C-terminus and is capable of retaining the γ-TURC-containing microtubule-nucleating complex (gamma-TURC) at the N-terminus [56]. The localization of ODF2 is described as being closest to the surface of the centriolar cylinder and critical for the formation of subdistal appendages. Originally, it has been proposed that ODF2 participates in the formation of both distal and subdistal appendages [64], but this has been disputed for distal appendages by Tanos *et al*. in 2013 [65].

Summarizing the accumulated data, Huang *et al*. in 2017 [29] described two new subdomain appendages of both CCDC120 and CCDC6. They further dissected a hierarchical assembly scheme for subdistal appendages. According to this scheme, ODF2 acts upstream to initiate the assembly [26,64]; TCHP mediates the interaction between ODF2 and ninein [66]; CEP170 can be recruited by ninein [67]. CCDC120 is recruited to subdistal appendages by ODF2 and recruits both CEP170 and ninein to subdistal appendages through two distinct domains [29], CCDC68 competes with CCDC120 in recruiting CEP170 [29].

In the past decade, the proteins of the distal appendages of the CEP family—CEP164 [68], CEP89 [69], CEP83, and the proteins SCLT1 and FBF1 [65] have been characterized. It has been shown that suppression of CEP164 protein expression leads to blockage of ciliogenesis [68]. The suppression of each of the proteins CEP89, CEP83, SCLT1 and FBF1 blocked the appearance of distal appendages on the surface of the centrioles and ciliogenesis [65]. Based on their experimental data on the differential reduction of various proteins, the authors constructed a hierarchical scheme in which CEP83 is the first protein appearing on the centriole surface, the presence of CEP89 and SCLT1 proteins depends on the presence of CEP83 on the centriole. From the appearance of SCLT1 depends on the accumulation on the centrioles of two more proteins of the distal appendages CEP164 and FBF1 [65].

Depletion of CEP83 did not affect the localization of ODF2, which the authors interpreted as the independence of the regulation of assembly of the distal and subdistal appendages [65]. We note that for electron microscopists who observed the distal and the subdistal appendages both components of the centrosome in cells at different stages of the cell cycle and in different experimental situations, this conclusion was obvious in advance.

## 6. Conclusion

The important take-home message of this review is that distal and subdistal appendages differ quantitatively, morphologically, biochemically and functionally. Common to these structures is their location on the older mother centriole; although in cells with very high MT nucleation activity subdistal appendages may develop on younger centrioles, as well in leucocytes of axolotl [18] and granulocytes of human blood [48].

**Figure 5.**
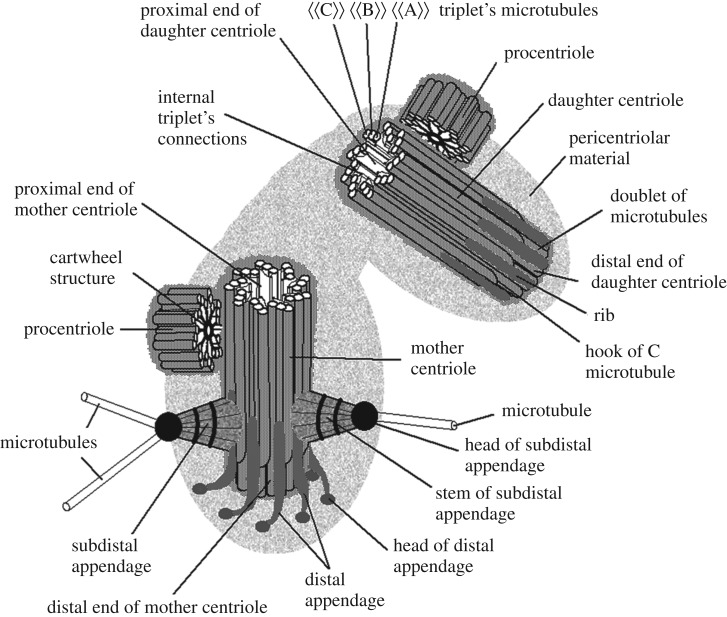
A scheme of centrosome in S-phase of cell cycle. Two subdistal appendages are shown here what is typical for epithelial pig kidney (PK) embryo cells. Serial sections cells of this cell line were used for model construction. Near the distal pole the MT ‘C’ terminates earlier than MT ‘A’ and MT ‘B’. In the region of triplet to doublet transition MT ‘C’ is uncomplete; this structure is called as ‘hook’. There are no distal appendages on the daughter centriole, but electronically dense plats (called ‘ribs’) are there on each triplet. Other terms are explained in the text. From [70,71] with small modifications.

The differences between these structures are significant: (i) the distal appendages are located centrally symmetrically (symmetry of the 9th order), while the subdistal (as a rule) are not symmetrical; (ii) the distal appendages are rigidly attached to the distal end of the centriole at the same level while the subdistal appendages can be located along the entire length of the centriole down to its proximal end; (iii) the number of distal appendages is always 9 (one for each triplet of the centriolar cylinder), but the number of subdistal appendages can vary from 0 to 14 depending on the type of cells and their functional state; (iv) two types of appendages are composed of different sets of proteins; and (v) two types of appendages are functionally different (figure 5).

## Data accessibility

This article has no additional data.
